# Impact of prenatal screening on the prevalence of Down syndrome in Slovenia

**DOI:** 10.1371/journal.pone.0180348

**Published:** 2017-06-30

**Authors:** Gorazd Rudolf, Nataša Tul, Ivan Verdenik, Marija Volk, Anamarija Brezigar, Nadja Kokalj Vokač, Nataša Jeršin, Bernarda Prosenc, Tanja Premru Sršen, Borut Peterlin

**Affiliations:** 1 Clinical Institute of Medical Genetics (CIMG), University Medical Centre Ljubljana, Ljubljana, Slovenia; 2 Department of Perinatology, Division of Gynaecology and Obstetrics, University Medical Centre Ljubljana, Ljubljana, Slovenia; 3 Medgen, Outpatient Clinic for Medical Genetics, Ljubljana, Slovenia; 4 Laboratory for Medical Genetics, University Medical Centre Maribor, Maribor, Slovenia; Holbæk Hospital, DENMARK

## Abstract

**Objectives:**

To evaluate the impact of prenatal screening and genetic testing for trisomy 21 (T21) on the prevalence of T21 in Slovenia.

**Design and setting:**

Data about all prenatally and postnatally confirmed cases of T21 in Slovenia between 1981 and 2012 were collected retrospectively from all genetic laboratories in Slovenia. The expected number of babies with T21 according to maternal age was calculated.

**Main outcome measures:**

The primary outcomes measures were number of fetuses and newborn infants with T21 diagnosed prenatally and postnatally and the impact of advances in screening and genetic diagnostics on the prevalence of newborns with T21 in Slovenia.

**Results:**

Despite a significantly increased mean maternal age from 25.4 years in year 1981 to 30.3 years in year 2012 the prevalence of newborn infants with T21 was 0.51 per 1000 births compared to 0.55 per 1000 births, respectively. The prevalence of prenatally diagnosed cases increased from 0.03 per 1000 births to 2.06 per 1000. The detection rate of T21 in year 2012 was 78,9%. The total number of prenatal invasive procedures (chorionic villous sampling and amniocenteses) carried out during that period was rising until 2002, since when it is stable at around 7%.

**Conclusion:**

The advancement and implementation of screening tests and prenatal diagnostic procedures in Slovenia caused an important improvement in the efficiency of the prenatal detection of T21.

## Introduction

Down’s syndrome (T21) is the most common aneuploidy compatible with life and the most common cause of congenital developmental delay and represents an important public health challenge [1,2].

The average maternal age is increasing in Europe since the late 1970s. An increasing number of newborn infants with T21 is expected, since the prevalence of T21 is associated with maternal age. Prenatal screening and termination of affected pregnancies could counteract this effect, although this varies between countries depending on the policy, provision and uptake of prenatal screening [3,4,5].

Early prenatal diagnosis of T21 and up-to-date information given by health-care providers in the process of non-directive counselling helps the parents to understand the condition. Medical professionals should provide necessary support during the prenatal diagnostic process and respect the autonomy of the parents and their informed decision about the pregnancy continuation or not [3,4,5].

Prior to 1996 it was maternal age and abnormalities detected by an ultrasound scan that were the only indications for prenatal diagnostic procedure in Slovenia. They were followed by screening for T21 using the triple test, which was introduced in 1996 and by nuchal translucency and combined test (double test) in 2000 and 2002 followed. In 2006 the triple test was replaced with the quadruple test. The combined screening test consists of ultrasound nuchal translucency (NT) measurements and the use of serum marker levels in maternal blood in the first trimester of pregnancy, which, combined with maternal age, is used to assign the risk of the pregnancy having T21. Women with the estimated risk above the cut-off (e.g. 1 in 300 for nuchal translucency or 1 in 190 for the quadruple test) are then offered an invasive diagnostic procedure—chorionic villous sampling (CVS) or amniocentesis. The first amniocentesis in Slovenia was performed in 1981 and first CVS in 1985. Slovenia´s health insurance covers a T21 screening in pregnant women aged 35 to 37 years and an invasive diagnostic procedure in pregnant women with the increased risk for T21, e.g. advanced maternal age (≥37 years), positive screening test, and ultrasound markers or abnormalities.

Despite similar T21 screening recommendations in the EU, there are differences in the T21 uptake and are a result of differences in health and social care legislation and funding, and, in addition, cultural and personal factors across these countries [4,5]. In countries where all pregnant women are offered screening for T21, they report on 75–90% detection rate for T21 [3,4,5], while in countries where screening is less fully implemented, the detection can be much lower (10–35%) [5].

The main purpose of our study was to examine the impact of screening for T21 in Slovenia on the prevalence of T21 in newborns over the last 30 years.

## Methods

We collected all the prenatal and postnatal recorded cases of T21 confirmed by karyotyping for the period between 1981 and 2012 in Slovenia (S1 Table).

Data on prenatal detection was collected from three cytogenetic laboratories in Slovenia which are involved in prenatal diagnosis: University Medical Centre (UMC) Ljubljana, UMC Maribor, and Medgen Ljubljana. Pregnant women had prenatal diagnostic testing because of advanced maternal age, positive screening test for DS, or anomalies detected by ultrasound. Data collected from a primary source were validated with information from records of the Ethical Commission for pregnancy termination and of the Slovenian National Perinatal Information System (NPIS). NPIS registers all deliveries in Slovenia at ≥ 22 weeks of pregnancy or when the fetus weights ≥ 500 g since 1987, accompanied by data on screening and invasive tests.

Data on postnatally detected cases were collected from the above mentioned laboratories and completed by information from the Institute for Public Health of Slovenia.

From this database we extracted maternal age and calculated the expected number of liveborns with T21 adjusted for non-viability according to Morris et al. [6].

## Results

### 1. Indications for invasive tests

The introduction of different screening tests for T21 increased the proportion of T21 diagnosed prenatally from 6,25% in 1981 to 78,9% in 2012.

**Fig 1:**
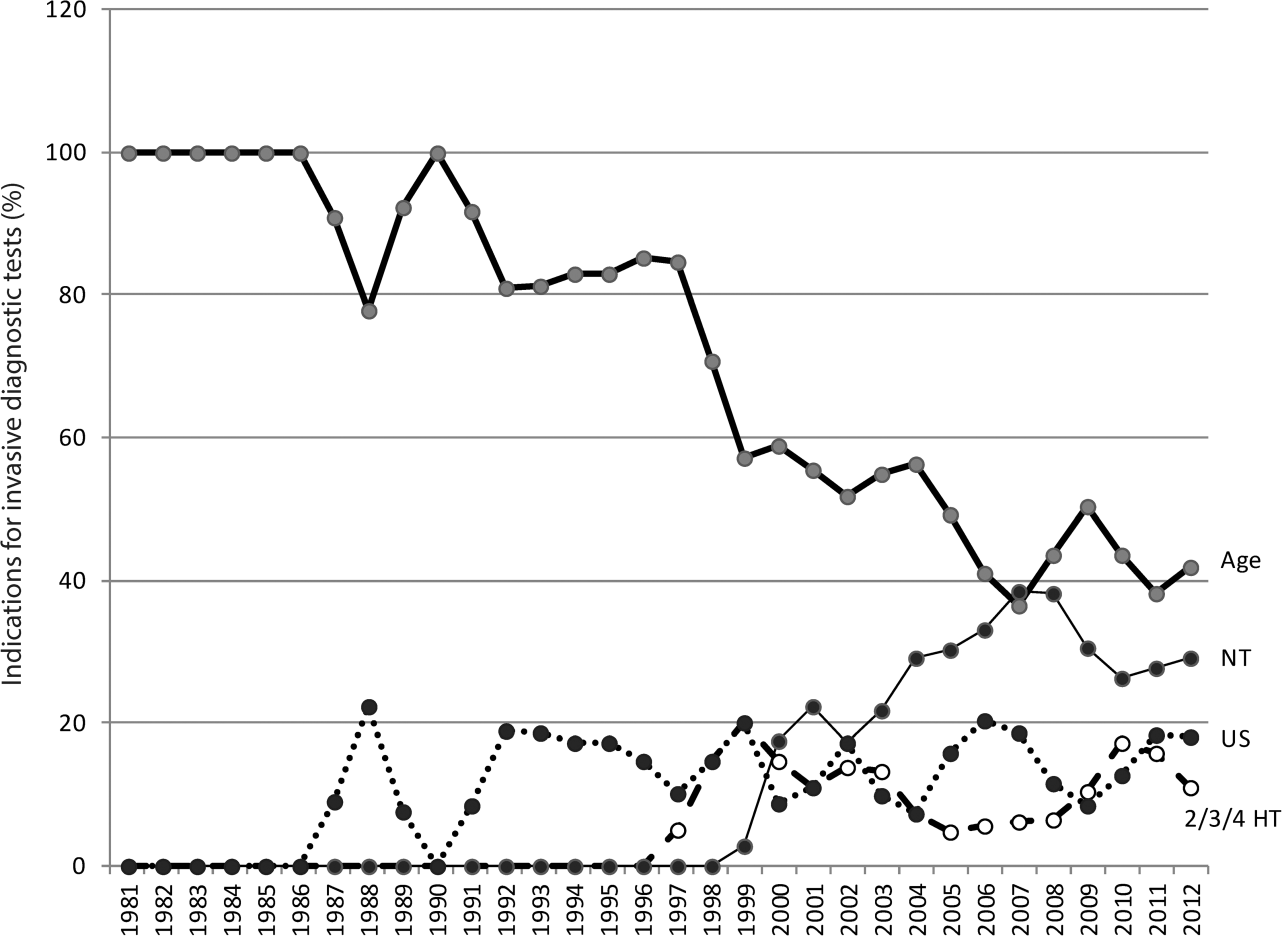
Indications for prenatal invasive diagnostic tests for the period 1981–2012. NT–nuchal translucency measurment, 2/3/4 HT–combined, triple or quadriple hormonal test, US–ultrasound abnormalities.

Fig 1 shows the relation (proportion) between advanced maternal age and different screening tests as an indication for prenatally diagnosed T21.

In 2012, 46% of prenatally identified fetuses with T21 were diagnosed because of advanced maternal age, 38% because of a screening test, and 16% because of ultrasound findings (congenital anomalies and soft markers).

Until the introduction of the screening tests, when the main indication for prenatal genetic testing was advanced maternal age, the number of fetuses with T21 diagnosed prenatally was 5 or less cases per year.

After the introduction of the triple test, nuchal translucency measurement (NT), combined and quadruple screening tests and, moreover, because of the awareness of the existence of screening tests among health care professionals and women, the number of prenatally diagnosed cases is rising constantly. Nevertheless, in 2012 it is advanced maternal age that still represents the important indication for invasive tests in Slovenia (40%).

In 2012 nearly 80% of all Slovenian pregnant women underwent screening by NT and additional 3% by biochemical tests.

### 2. Total prevalence and the proportion of prenatally diagnosed T21

**Fig 2:**
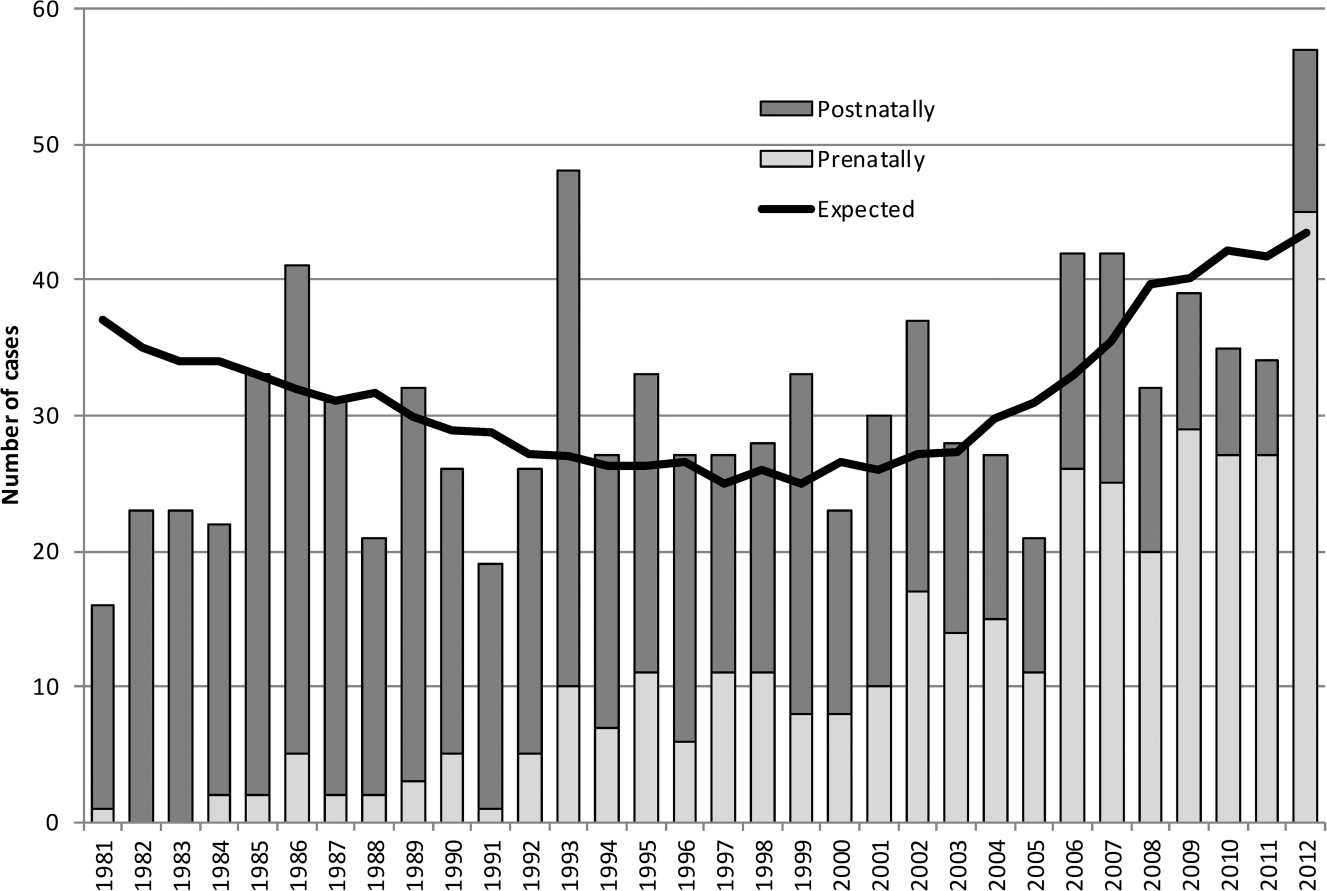
Numbers of total, prenatally diagnosed, newborn, and expected liveborn cases with T21 in Slovenia, 1981–2012.

The total prevalence of T21 increased from 0.54 per 1000 births in 1981 to 2.61 per 1000 in 2012. These values include live births and stillbirths diagnosed postnatally, and outcomes after prenatal diagnoses (terminations, recorded fetal losses, and continued pregnancies). Values are not adjusted for non-viable cases. The prevalence of newborn infants with T21 was 0.55 per 1000 births in year 2012, compared to 0.51 per 1000 births in year 1981. The proportion of cases diagnosed prenatally increased from 6,25% in year 1981 to 78,9% in year 2012 (Fig 2).

### 3. Prenatal invasive diagnostic procedure rate

**Fig 3:**
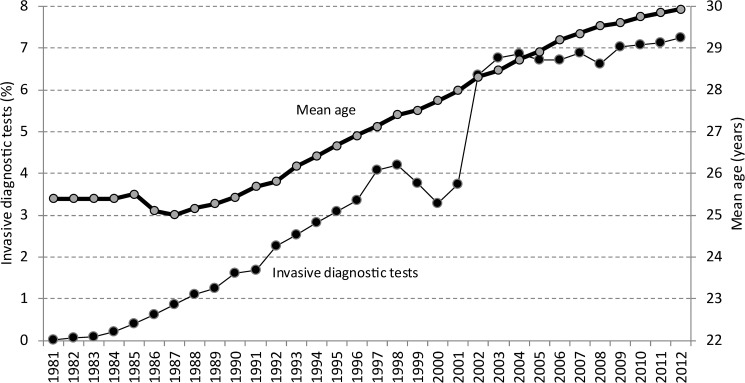
Mean maternal age and invasive diagnostic tests for the period between1981-2012.

The number of prenatal procedures (chorionic villous samplings or amniocenteses) increased steadily from 7 in 1981 (0.24 per 1000) to 1581 (72.3 per 1000) in 2012 (Fig 3). The number of chorionic villous samplings increased from 3 in 1985 to 322 in 2012, while the number of amniocenteses increased from 95 in 1985 to 1259 in 2012. This corresponds to an increase in the ratio of chorionic villous samplings compared to amniocentesis from 0.03 (year 1985) to 0.26 (year 2012).

## Discussion

This study maps the changes in the prevalence of newborn infants with T21 with the advancement of prenatal diagnostic methods during the last 30 years in Slovenia.

Prior to 1996, maternal age alone was used as a primary screening test with later advances in screening methods. Nevertheless, in 2012, 46% of all prenatally diagnosed fetuses with T21 are discovered because of the advanced maternal age, followed by combined screening tests and nuchal translucency measurements, whereas the smallest T21 proportion is contributed by fetal anomalies detected by an ultrasound (Fig 1). Across Europe the screening policy and uptake vary considerably by both prenatal diagnosis rate and types of screening tests performed [5]. Advanced maternal age represents a much lower indication rate for prenatal diagnosis in countries, where ultrasound and biochemical screening have been introduced on a population-wide basis [5]. We believe that the main reason for the persisting high rate of advanced maternal age as an indication for prenatal diagnosis in Slovenia is the current screening policy, since T21 screening is, at the moment, available on a patient self-pay basis for pregnant women aged 34 years or less.

Recently, cell free fetal DNA test (cffDNA) for fetal aneuploidy has been implemented in most developed countries [7,8]. In Slovenia, cffDNA has been available since 2012 on a patient self-pay basis, but the cumulative data summarizing the high-risk pregnancies and their outcomes are not at hand as of yet.

In Europe there are significant geographic inequalities in both total and live birth prevalence of T21 [5]. The variation in total prevalence is mainly due to the large variation in maternal age profile of European countries. On the other hand, the differences in the pregnancy termination rate after prenatal diagnosis of T21 between countries pose important geographical variation in live birth prevalence of T21 [5]. More than a three-fold higher live birth prevalence of T21 is observed in countries with legal restrictions of termination of pregnancy after a particular gestational age [5]. The total prevalence of T21 in Slovenia has increased from 0.54 per 1000 births in 1981 to 2.61 per 1000 in 2012. The increase in the total prevalence of T21 has been predicted by demographic analyses and results from the tendency to postpone family planning and the rising proportion of pregnant women aged more than 35 years, which would have more than tripled the number of births with T21 in our country. In addition, the observed mean maternal age has increased from 25.4 in year 1981 to 30.3 in 2012.

On the other hand, the observed prevalence of newborn infants with T21 should decrease because of prenatal screening, diagnostic tests, and pregnancy termination. The improvements in the T21 screening tests, accompanied by ultrasound imaging and genetic testing allowing for an earlier diagnosis of T21, have caused the number of newborns with T21 to decrease on the one hand, and increased the number of cases with trisomy 21 detected prenatally on the other. According to experiences in the developed countries the main factors influencing the prenatal detection rate of T21 are the promotion of prenatal screening, keeping pregnant women informed, and the availability of T21 screening to all pregnant women with invasive diagnostic procedure offered at a younger maternal age [9]. Denmark reports very high T21 uptake rates, since all pregnant women are given information about screening methods in pregnancy and, if desired, they are offered a combined risk assessment for T21 in the first trimester. Additionally, women aged 35 or more are offered an invasive diagnostic test with CVS or amniocentesis [1].

In Slovenia the prevalence of newborn infants with T21 was 0.55 per 1000 births in 2012 compared to 0.51 per 1000 births in 1981. The 2012 prevalence in Slovenia is one of the lowest among European countries [5]. We believe that a very high rate of pregnancy termination after prenatal diagnosis of T21 in Slovenia is an important factor for the low prevalence. The law permits medical termination of pregnancy even at an advanced gestation for medical problems which would severely compromise the quality of life. The majority of women with prenatally diagnosed T21 in Slovenia decide to terminate the pregnancy.

The proportion of cases diagnosed prenatally increased from 6,25% in 1981 to 78,9% in 2012, which is a significant achievement according to the current screening policy in Slovenia.

Similar results have been reported in many EU countries [5]. For example, in Hungary, the proportion of prenatally diagnosed cases increased from zero in 1970 to 44,3% in 1999, furthermore, the birth prevalence of T21 decreased by 57% [10]. Another study from the Paris Registry of Congenital Malformations based on 1981–2000 data has found an approximate 5% increase in the total prevalence of T21 and a 3% decrease in live birth prevalence per year. However, not all countries observed changes when introducing T21 screening; in the Netherlands, for example, a full programme for T21 screening has been implemented, yet they report on less than a 30% uptake rate [4]. Additionally, in the North American registers (Canada Alberta, Atlanta USA, Mexico) an increase in children with T21 has been observed, which was mainly due to fewer terminations of pregnancy with T21 [11].

The number of prenatal diagnostic procedures in Slovenia has steadily increased from 1980 until 2002, and has been on the rise even in the last years when all the screening tests have been available. In the first years this rise was mainly a result of the implementation and better accessibility of diagnostic procedures, and later due to the introduction of different screening tests. In contrast, a Danish study observed that the total number of invasive procedures had decreased by half in years 2000 to 2006 after the implementation of a screening policy that should be available to all pregnant women [1]. One of the reasons for the persistent high number of prenatal diagnostic tests in Slovenia is probably advanced maternal age, which is still the sole indication for prenatal testing in conjunction with increased mean maternal age.

In summary, the implementation of screening tests and prenatal diagnostic procedures has caused an important improvement of the efficiency in the prenatal detection of T21 in Slovenia.

## Supporting information

S1 Table Number of prenatally diagnosed fetuses with T21 and newborns with T21 in Slovenia for the period 1981–2012. (DOCX)
