# Supplementary material for: Impact of prenatal screening on the prevalence of Down syndrome in Slovenia
Source: PLoS One. 2017 Jun 30;12(6):e0180348. doi: 10.1371/journal.pone.0180348 (PMC5493396; doi:10.1371/journal.pone.0180348)
Supplement: S1 Table — (DOCX) [file pone.0180348.s001.docx]

| Year | Number of prenatally  diagnosed T21 | Newborns with T21 |
| --- | --- | --- |
| 1981 | 1 | 15 |
| 1982 | 0 | 23 |
| 1983 | 0 | 23 |
| 1984 | 2 | 20 |
| 1985 | 2 | 31 |
| 1986 | 5 | 36 |
| 1987 | 2 | 29 |
| 1988 | 2 | 19 |
| 1989 | 3 | 29 |
| 1990 | 5 | 21 |
| 1991 | 1 | 18 |
| 1992 | 4 | 21 |
| 1993 | 10 | 38 |
| 1994 | 7 | 20 |
| 1995 | 11 | 22 |
| 1996 | 6 | 21 |
| 1997 | 10 | 16 |
| 1998 | 11 | 17 |
| 1999 | 8 | 25 |
| 2000 | 8 | 15 |
| 2001 | 10 | 20 |
| 2002 | 17 | 20 |
| 2003 | 14 | 14 |
| 2004 | 15 | 12 |
| 2005 | 10 | 10 |
| 2006 | 26 | 16 |
| 2007 | 24 | 17 |
| 2008 | 20 | 12 |
| 2009 | 28 | 10 |
| 2010 | 27 | 8 |
| 2011 | 27 | 7 |
| 2012 | 45 | 12 |
| total | 361 | 617 |

**S1 Table. Number of prenatally diagnosed fetuses with T21 and newborns with T21 in Slovenia for the period 1981-2012.**
